# Evaluation of the Coverage of 3 Antibiotic Regimens for Neonatal Sepsis in the Hospital Setting Across Asian Countries

**DOI:** 10.1001/jamanetworkopen.2019.21124

**Published:** 2020-02-12

**Authors:** Julia A. Bielicki, Mike Sharland, Paul T. Heath, A. Sarah Walker, Ramesh Agarwal, Paul Turner, David A. Cromwell

**Affiliations:** 1 Paediatric Infectious Diseases Research Group, Institute of Infection and Immunity, St George’s University of London, London, United Kingdom; 2 Department of Health Services Research and Policy, London School of Hygiene and Tropical Medicine, London, United Kingdom; 3 Paediatric Pharmacology and Paediatric Infectious Diseases, University of Basel Children’s Hospital, Basel, Switzerland; 4 Medical Research Council Clinical Trials Unit at University College London, London, United Kingdom; 5 Department of Paediatrics, All India Institute of Medical Sciences, New Delhi, India; 6 Centre for Tropical Medicine and Global Health, Nuffield Department of Medicine, University of Oxford, United Kingdom; 7 Cambodia Oxford Medical Research Unit, Siem Reap, Cambodia

## Abstract

**Importance:**

High levels of antimicrobial resistance in neonatal bloodstream isolates are being reported globally, including in Asia. Local hospital antibiogram data may include too few isolates to meaningfully examine the expected coverage of antibiotic regimens.

**Objective:**

To assess the coverage offered by 3 antibiotic regimens for empirical treatment of neonatal sepsis in Asian countries.

**Design, Setting, and Participants:**

A decision analytical model was used to estimate coverage of 3 prespecified antibiotic regimens according to a weighted-incidence syndromic combination antibiogram. Relevant data to parameterize the models were identified from a systematic search of Ovid MEDLINE and Embase. Data from Asian countries published from 2014 onward were of interest. Only data on blood culture isolates from neonates with sepsis, bloodstream infection, or bacteremia reported from the relevant setting were included. Data analysis was performed from April 2019 to July 2019.

**Exposures:**

The prespecified regimens of interest were aminopenicillin-gentamicin, third-generation cephalosporins (cefotaxime or ceftriaxone), and meropenem. The relative incidence of different bacteria and their antimicrobial susceptibility to antibiotics relevant for determining expected concordance with these regimens were extracted.

**Main Outcomes and Measures:**

Coverage was calculated on the basis of a decision-tree model incorporating relative bacterial incidence and antimicrobial susceptibility of relevant isolates. Data on 7 bacteria most commonly reported in the included studies were used for estimating coverage, which was reported at the country level.

**Results:**

Data from 48 studies reporting on 10 countries and 8376 isolates were used. Individual countries reported 51 (Vietnam) to 6284 (India) isolates. Coverage varied considerably between countries. Meropenem was generally estimated to provide the highest coverage, ranging from 64.0% (95% credible interval [CrI], 62.6%-65.4%) in India to 90.6% (95% CrI, 86.2%-94.4%) in Cambodia, followed by aminopenicillin-gentamicin (from 35.9% [95% CrI, 27.7%-44.0%] in Indonesia to 81.0% [95% CrI, 71.1%-89.7%] in Laos) and cefotaxime or ceftriaxone (from 17.9% [95% CrI, 11.7%-24.7%] in Indonesia to 75.0% [95% CrI, 64.8%-84.1%] in Laos). Aminopenicillin-gentamicin coverage was lower than that of meropenem in all countries except Laos (81.0%; 95% CrI, 71.1%-89.7%) and Nepal (74.3%; 95% CrI, 70.3%-78.2%), where 95% CrIs for aminopenicillin-gentamicin and meropenem were overlapping. Third-generation cephalosporin coverage was lowest of the 3 regimens in all countries. The coverage difference between aminopenicillin-gentamicin and meropenem for countries with nonoverlapping 95% CrIs ranged from −15.9% in China to −52.9% in Indonesia.

**Conclusions and Relevance:**

This study’s findings suggest that noncarbapenem antibiotic regimens may provide limited coverage for empirical treatment of neonatal sepsis in many Asian countries. Alternative regimens must be studied to limit carbapenem consumption.

## Key Points

**Question:**

What is the antibiotic coverage offered by empirical neonatal sepsis treatment with aminopenicillin-gentamicin, third-generation cephalosporins (cefotaxime or ceftriaxone), and meropenem in Asian countries?

**Findings:**

In this decision analytical model based on a decision tree, 8376 isolates from 10 countries were used to estimate coverage. Meropenem generally had the highest coverage (from 64.0% in India to 90.6% in Cambodia) followed by aminopenicillin-gentamicin (from 35.9% in Indonesia to 81.0% in Laos) and cefotaxime or ceftriaxone (from 17.9% in Indonesia to 75.0% in Laos); in all countries except Laos and Nepal, meropenem coverage was higher than that of the other 2 regimens.

**Meaning:**

The findings suggest that noncarbapenems may provide limited empirical neonatal sepsis coverage in many Asian countries.

## Teaser

This decision analytical model assesses the coverage offered by 3 antibiotic regimens for empirical treatment of neonatal sepsis in 10 Asian countries.

## Introduction

Although overall maternal and child mortality have substantially declined worldwide since the early 2000s, neonatal mortality associated with bacterial infection has remained high, with nearly half a million estimated annual deaths due to neonatal sepsis.^1^ Most of these deaths occur in low- and middle-income countries (LMICs), including many thousands in Asia.^2^

In a recent prospective cohort study^3^ of more than 13 500 live births in India, the case-fatality rate of culture-positive neonatal sepsis episodes was nearly 50%. Recent systematic reviews^4,5,6,7^ indicate a high level of bacterial resistance to World Health Organization (WHO)–recommended empirical treatment regimens for serious neonatal and pediatric infections in LMICs, especially in bloodstream isolates. Globally, antimicrobial resistance is estimated to be implicated in up to one-third of neonatal sepsis deaths annually.^8^

Clinicians and guideline-setting bodies can be assisted in selecting optimal empirical antibiotic regimens by knowing the coverage of alternative regimens.^9^ Regimen coverage refers to the proportion of infection episodes that would be treated by the regimen at a stage when the causative pathogen is not yet known, therefore incorporating the frequencies of different causative bacteria and their resistance patterns. Several techniques are available to estimate coverage. One example is the weighted-incidence syndromic combination antibiogram (WISCA),^9,10,11^ which estimates coverage by accounting for the relative incidence of different bacteria and their resistance patterns for a specific infection syndrome, in this case neonatal sepsis. Coverage can be estimated for both single-drug and combination treatment regimens.

International guidelines provide recommendations for the empirical antibiotic treatment of neonatal bacterial infections and should aim to provide adequate coverage in target settings, especially LMICs.^12^ The objective of this decision analytical model study was, therefore, to evaluate the coverage offered by 3 prespecified antibiotic regimens according to WISCAs and focusing on Asia, a region with a high prevalence of bacterial resistance.

## Methods

We estimated coverage using data on antimicrobial resistance that were used to create WISCAs for each country with reported data,^9^ as identified by a systematic review of the literature. Because only published data were used in the analysis, no formal ethical review was required according to guidance by the NHS Health Research Authority. This study follows the Consolidated Health Economic Evaluation Reporting Standards (CHEERS) reporting guideline, because it is broadly applicable to any decision-model based analyses (eAppendix in the Supplement).^13^

### Regimens Selected for Coverage Estimation

The 3 regimens evaluated in this study were aminopenicillin-gentamicin (WHO-recommended first-line treatment; alternatives, benzylpenicillin or cloxacillin plus gentamicin), third-generation cephalosporins (WHO-recommended second-line treatment, assumed to be cefotaxime or ceftriaxone, not ceftazidime), and meropenem.^12^ The last regimen was evaluated because it has now been reported to be the most commonly used empirical treatment in LMICs for neonatal sepsis.^14^

### Identification of Relevant Data for Parameter Estimation

A systematic search of the literature was conducted in Ovid MEDLINE and in Embase on January 23, 2019. Using both free-text and MeSH terms, publications on “sepsis” and “antibiotic resistance” and (“neonates” or infants”) and “Asia” were identified (eAppendix in the Supplement). Given increasing antimicrobial resistance, and to obtain contemporaneous estimates, we arbitrarily limited the search to articles published from 2014 onward. No additional limits were applied. Studies were reviewed against prespecified eligibility criteria, and data were extracted using a standardized prepiloted form implemented in REDCap^15^ (eAppendix in the Supplement).

Extracted data for WISCA calculation included information on the total number of bacterial isolates from relevant blood cultures, the number of isolates of specific bacterial species or genera, the number of isolates tested for susceptibility to the antibiotics relevant for establishing coverage offered by the prespecified regimens of interest, and the number of isolates found to be susceptible to these antibiotics. We excluded bacteria known to frequently represent contamination rather than true infection, most importantly coagulase-negative staphylococci.^16^ The exclusion of coagulase-negative staphylococci is likely to result in the overestimation of coverage for β-lactam–based regimens because of very high expected rates of methicillin resistance of 66% to more than 90%.^17,18^

### Estimation of WISCA Parameters

Tables containing the parameter values required for coverage estimation were created by country and regimen. The relative incidence parameters were based only on bacteria reported as contributing to neonatal sepsis in more than 50% of the eligible studies. This meant that estimated coverage was based on the most important and frequent pathogens identified in blood cultures from neonates in the target region. Including rare pathogens within the WISCA would have a minimal impact on the estimated coverage, and including those likely to be contaminants or unusual pathogens (potentially observed as part of unidentified outbreaks) could introduce substantial bias. For the bacteria identified in this way, their relative incidence was based on the frequency reported in the studies. Similarly, regimen susceptibility was derived directly from reported data with the number of tested isolates representing the denominator. Details of the assumptions for determining susceptibility of pathogens to each regimen are provided in the eAppendix in the Supplement.

### Statistical Analysis

Regimen coverage was estimated using a previously described Bayesian WISCA.^9^ This approach has various advantages. It addresses the typical clinical approach of treating an infection syndrome, often with incomplete knowledge about the frequency of causative bacteria and their susceptibilities. The Bayesian WISCA also explicitly deals with intrinsic resistance and handles imprecision attributed to a small sample size or incomplete susceptibility testing data.

In brief, the WISCA gives the expected levels of therapeutic coverage for an antibiotic regimen—in our case, regimens used to treat neonates with sepsis. The WISCA can be represented as a decision tree (eFigure 1 in the Supplement). Combining the probabilities along the regimen tree branches generates coverage estimates from relative bacterial incidence and proportions of each included pathogen susceptible to the antibiotic regimen. In essence, the WISCA is a weighted mean of the susceptibilities of the bacteria, with the weights defined by their relative incidence.

The observed data on pathogen incidence and their susceptibility to the 3 regimens were combined with an appropriate Bayesian prior distribution that corresponded to our prestudy beliefs about these parameters. We had no strong prior belief about the relative incidence of the pathogens or for the majority of what level of susceptibility there might be within a country, and a noninformative prior was used in these cases. However, in some circumstances, specific pathogens were expected to have intrinsic resistance to the regimen and, consequently, not to have susceptibility regardless of reported susceptibility testing results.^19,20^ In these situations, an informative prior was used to dominate the observed data. On the basis of European Committee for Antimicrobial Susceptibility Testing (EUCAST) recommendations,^19,20^ enterococci, as well as *Acinetobacter* species and *Pseudomonas* species, were assumed to be intrinsically resistant to recommended third-generation cephalosporins and therefore not susceptible to third-generation cephalosporins.

The value of the pathogen incidence and pathogen regimen-susceptibility parameters were defined as probability distributions to reflect the uncertainty in their respective values. The relative incidence of pathogens was modeled using a Dirichlet distribution, and the susceptibility parameters were defined as beta distributions; 95% credible intervals (95% CrIs) for the coverage estimates were calculated using Monte Carlo simulations, based on 1000 runs (eAppendix in the Supplement). All modeling was undertaken using Stata statistical software version 13.1 (StataCorp) and Excel spreadsheet software version 2010 (Microsoft Corp). Data analysis was performed from April 2019 to July 2019.

## Results

### Description of Data Set

The literature review included data from 48 publications (eFigure 2 in the Supplement) representing 52 centers in 10 Asian countries (1 center in Cambodia, 5 in China, 33 in India, 1 in Indonesia, 1 in Laos, 1 in Malaysia, 6 in Nepal, 2 in Pakistan, 1 in Taiwan, and 1 in Vietnam). Of the 52 centers, 34 were university or tertiary hospitals, 10 were nonteaching or district hospitals (9 in India and 1 in China), and 8 were maternity or pediatric hospitals (1 in Cambodia, 2 in China, 4 in Nepal, and 1 in Vietnam).

Ten articles were published in 2014, 13 in 2015, 10 in 2016, 8 in 2017, 6 in 2018, and 1 in 2019. For 32 of 48 publications, the observation period started in 2010 or later, with the earliest start date being January 1, 1990 (eTable 1 in the Supplement). Five publications did not report calendar dates for their observation period, but 4 of 5 indicated its duration. The median observation period was 2 years, with the shortest and longest periods being 2 months and 12 years, respectively.

Most publications (33 of 48) reported on bloodstream isolates from neonates with clinical community-acquired or nosocomial sepsis. Another 12 publications based reporting on microbiologically defined bacteremia. Only 4 publications focused on either nosocomial or community-acquired infections (2 each). Reporting of information on sample processing, including species identification, antibiotic susceptibility testing methods, and interpretive guidelines, was variable (eTable 2 in the Supplement).

### Reported Bloodstream Isolates

Individual publications included between 15 and 2112 isolates, with a median of 98 isolates (eTable 3 in the Supplement). The following bacteria were most frequently reported as contributing to neonatal sepsis or bacteremia: *Escherichia coli* (46 of 48 publications), *Klebsiella* species and *Staphylococcus aureus* (45 of 48 publications each), *Pseudomonas* species (35 of 48 publications), *Acinetobacter* species (32 of 48 publications), *Enterobacter* species (26 of 48 publications), and *Enterococcus* species (25 of 48 publications). In addition, coagulase-negative staphylococci were reported in 40 of 48 publications. All other bacteria, including *Citrobacter* species and *Streptococcus agalactiae*, were reported in less than one-half of the publications. On the basis of the prespecified criteria, *E coli*, *Klebsiella* species, *S aureus*, *Pseudomonas* species, *Acinetobacter* species, *Enterobacter* species, and *Enterococcus* species were selected for antibiotic regimen coverage estimation.

### Parameter Values: Isolates Reported and Susceptibility

In total, 11 467 isolates were reported, with the greatest number coming from India (6284), China (2043), Pakistan (1875), and Nepal (640) (Table 1). Given the small number of reported isolates from Taiwan (36) and Malaysia (29), antibiotic regimen coverage was not estimated for these 2 countries. Most reported isolates (8584 of 11 467 [74.9%]) were from university or tertiary hospitals, with nonteaching or district hospitals contributing 11.5% (1319 of 11 467) and maternity or pediatric hospitals contributing another 13.6% (1564 of 11 467).

**Table 1.** Relative Incidence Data

Pathogen	Isolates, No. (%)^a^										
	Cambodia (n = 185)	China (n = 2043)	India (n = 6284)	Indonesia (n = 225)	Laos (n = 75)	Malaysia (n = 29)	Nepal (n = 640)	Pakistan (n = 1875)	Taiwan (n = 36)	Vietnam (n = 75)	Total (N = 11 467)
Contributing to WISCA											
* Escherichia coli*	25 (16)	300 (33)	671 (14)	0	8 (13)	6 (33)	50 (10)	976 (57)	11 (92)	2 (4)	2049 (24)
* Klebsiella* species	60 (39)	264 (29)	1065 (22)	49 (40)	9 (14)	1 (6)	45 (9)	159 (9)	1 (8)	18 (35)	1671 (20)
* Enterobacter* species	18 (11)	58 (6)	167 (3)	20 (17)	4 (6)	0	30 (6)	0	0	6 (12)	303 (4)
* Acinetobacter* species	16 (10)	27 (3)	992 (21)	21 (17)	2 (3)	0	63 (13)	0	0	17 (33)	1138 (14)
* Pseudomonas* species	6 (4)	53 (6)	430 (9)	31 (26)	1 (2)	1 (6)	25 (5)	199 (12)	0	4 (8)	750 (9)
* Staphylococcus aureus*	33 (21)	112 (12)	1235 (26)	0	37 (58)	10 (55)	261 (53)	388 (23)	0	4 (8)	2080 (25)
* Enterococcus* species	0	91 (10)	275 (6)	0	3 (5)	0	15 (3)	1 (<1)	0	0	385 (5)
Total reported during observation period											
Total contributing to WISCA	158 (85)	905 (44)	4835 (77)	121 (54)	64 (85)	18 (62)	489 (76)	1723 (92)	12 (33)	51 (68)	8376 (73)
Other (not contributing to WISCA)	27 (15)	1138 (56)	1449 (23)	104 (46)	11 (15)	11 (38)	151 (24)	152 (8)	24 (67)	24 (32)	3091 (27)
Coagulase-negative staphylococci (not contributing to WISCA)	0	741 (36)	980 (16)	63 (28)	0	0	137 (21)	28 (1)	0	23 (31)	1972 (17)

Abbreviation: WISCA, weighted-incidence syndromic combination antibiogram.

^a^ Percentages may not add to 100% because of rounding.

In total, 8376 isolates from 10 countries were used to estimate coverage. The proportion of reported isolates contributing to antibiotic regimen coverage estimation ranged from 91.9% (1723 of 1875) in Pakistan to 44.2% (905 of 2043) in China. Disregarding coagulase-negative staphylococci, the proportion of reported bacterial isolates contributing to coverage estimation ranged from 98.0% (51 of 52) in Vietnam to 69.5% (905 of 1302) in China.

Availability of susceptibility testing information for aminopenicillin-gentamicin coverage ranged from 68.8% (623 of 905) in China to 100% in Indonesia (Table 2). For third-generation cephalosporins, this was available for 100% in Cambodia and Indonesia and 76.5% (39 of 51) in Vietnam (Table 3). For meropenem, available susceptibility testing information ranged from 100% in Indonesia to 60.3% (295 of 489) in Nepal (Table 4).

**Table 2.** Susceptibility Testing and Susceptibility Data for Aminopenicillin Plus Gentamicin

Pathogen	No. of Isolates																										
	Cambodia			China			India			Indonesia			Laos			Nepal			Pakistan			Vietnam			Total		
	N	T	S	N	T	S	N	T	S	N	T	S	N	T	S	N	T	S	N	T	S	N	T	S	N	T	S
*Escherichia coli*	25	25	13	300	290	182	671	655	426	0	NA	NA	8	8	6	50	50	31	976	976	340	2	0	NA	2033	2004	998
*Klebsiella* species	60	60	10	264	256	193	1065	1026	402	49	49	3	9	9	7	45	42	23	159	159	36	18	11	2	1669	1612	676
*Enterobacter* species	18	18	8	58	20	11	167	154	42	20	20	18	4	0	NA	30	30	21	0	NA	NA	6	5	3	303	247	103
*Acinetobacter* species	16	0	NA	27	0	NA	992	930	226	21	21	11	2	0	NA	63	62	34	0	NA	NA	17	17	3	1138	1030	274
*Pseudomonas* species	6	0	NA	53	0	NA	430	422	238	31	31	9	1	0	NA	25	23	18	199	199	74	4	4	1	749	679	340
*Staphylococcus aureus*	33	33	32	112	56	31	1235	1142	655	0	NA	NA	37	37	37	261	227	195	388	88	63	4	3	3	2070	1586	1016
*Enterococcus* species	0	NA	NA	91	1	0	275	132	44	0	NA	NA	3	0	NA	15	15	12	1	0	NA	0	NA	NA	385	148	56

Abbreviations: N, total isolates; NA, not applicable; S, isolates identified as susceptible on testing; T, susceptibility testing available for regimen of interest.

**Table 3.** Susceptibility Testing and Susceptibility Data for Third-Generation Cephalosporins

Pathogen	No. of Isolates																										
	Cambodia			China			India			Indonesia			Laos			Nepal			Pakistan			Vietnam			Total		
	N	T	S	N	T	S	N	T	S	N	T	S	N	T	S	N	T	S	N	T	S	N	T	S	N	T	S
*Escherichia coli*	25	25	13	300	289	165	671	657	339	0	NA	NA	8	8	7	50	43	25	976	976	317	2	0	NA	2033	1998	866
*Klebsiella *species	60	60	4	264	251	122	1065	1031	346	49	49	2	9	9	6	45	42	12	159	159	52	18	11	1	1669	1612	545
*Enterobacter *species	18	18	1	58	20	14	167	167	59	20	20	17	4	0	NA	30	28	12	0	NA	NA	6	4	1	303	257	104
*Acinetobacter *species^a^	16	16	0	27	27	0	992	992	0	21	21	0	2	2	0	63	63	0	0	NA	NA	17	17	0	1138	1138	0
*Pseudomonas *species^a^	6	6	0	53	53	0	430	430	0	31	31	0	1	1	0	25	25	0	199	199	0	4	4	0	749	749	0
*Staphylococcus aureus*	33	33	32	112	56	31	1235	1142	655	0	NA	NA	37	37	37	261	227	195	388	88	63	4	3	3	2070	1586	1016
*Enterococcus *species^a^	0	NA	NA	91	91	0	275	275	0	0	NA	NA	3	3	0	15	15	0	1	1	0	0	NA	NA	385	385	0

Abbreviations: N, total isolates; NA, not applicable; S, isolates identified as susceptible on testing; T, susceptibility testing available for regimen of interest.

^a^ Not based on susceptibility testing because pathogen was assumed to be intrinsically resistant.

**Table 4.** Susceptibility Testing and Susceptibility Data for Meropenem

Pathogen	No. of Isolates																										
	Cambodia			China			India			Indonesia			Laos			Nepal			Pakistan			Vietnam			Total		
	N	T	S	N	T	S	N	T	S	N	T	S	N	T	S	N	T	S	N	T	S	N	T	S	N	T	S
*Escherichia coli*	25	24	24	300	289	289	671	439	379	0	NA	NA	8	0	NA	50	3	1	976	811	768	2	0	NA	2033	1566	1461
*Klebsiella* species	60	60	60	264	253	228	1065	882	667	49	49	49	9	0	NA	45	27	27	159	102	87	18	9	9	1669	1382	1127
*Enterobacter* species	18	18	17	58	20	20	167	157	122	20	20	19	4	0	NA	30	16	14	0	NA	NA	6	3	3	303	234	195
*Acinetobacter* species	16	16	14	27	0	NA	992	926	475	21	21	21	2	0	NA	63	7	3	0	NA	NA	17	16	15	1138	986	528
*Pseudomonas *species	6	5	5	53	0	NA	430	415	354	31	31	23	1	0	NA	25	0	NA	199	199	188	4	3	3	749	653	573
*Staphylococcus aureus*	33	33	32	112	56	31	1235	1142	655	0	NA	NA	37	37	37	261	227	195	388	88	63	4	3	3	2070	1586	1016
*Enterococcus* species^a^	0	NA	NA	91	91	0	275	275	0	0	NA	NA	3	3	0	15	15	0	1	1	0	0	NA	NA	385	385	0

Abbreviations: N, total isolates; NA, not applicable; S, isolates identified as susceptible on testing; T, susceptibility testing available for regimen of interest.

^a^ Not based on susceptibility testing because pathogen was assumed to be intrinsically resistant.

### Coverage Estimates at Country Level

Coverage was consistently lowest for third-generation cephalosporin monotherapy, with some variation across the individual countries, ranging from 56.6% (95% CrI, 52.2%-60.7%) in Nepal to 17.9% (95% CrI, 11.7%-24.7%) in Indonesia (Figure). Similarly, although meropenem had the highest estimated coverage in each country, the proportion of neonates for whom it would be effective empirical treatment varied considerably, from 90.6% (95% CrI, 86.2%-94.4%) in Cambodia to 64.0% (95% CrI, 62.6%-65.4%) in India (Figure). Aminopenicillin-gentamicin offered the second highest level of coverage within each country behind meropenem. Nonetheless, there was again considerable variability in country-level estimates, from 74.3% (95% CrI, 70.3%-78.2%) in Nepal to 35.9% (95% CrI, 27.7%-44.0%) in Indonesia (Figure).

**Figure.**
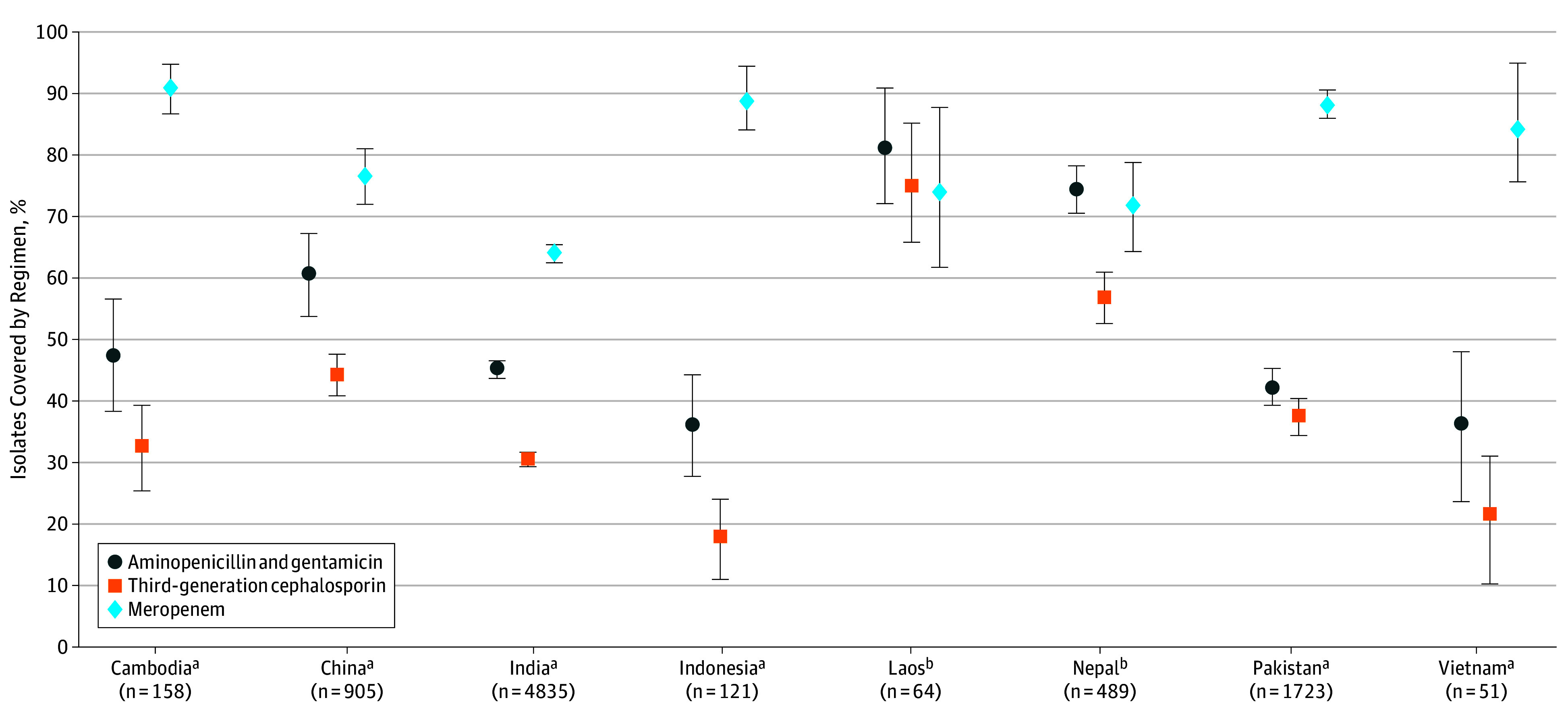
Coverage Estimates for 8 Asian Countries Point estimates are shown with 95% credible intervals, as denoted by error bars. Nonoverlapping 95% credible intervals indicate likely within-country differences in regimen coverage. Countries are shown together with the overall number of isolates used for estimating coverage. ^a^The highest coverage offered by meropenem was in Cambodia (90.6%), China (76.5%), India (64.0%), Indonesia (88.8%), Pakistan (88.1%), and Vietnam (84.1%). ^b^The highest coverage offered by aminopenicillin-gentamicin combination was in Laos (81.0%) and Nepal (74.3%).

Aminopenicillin-gentamicin coverage was higher than that offered by third-generation cephalosporins in China (60.6% [95% CrI, 54.2%-67.5%] vs 44.2% [95% CrI, 40.9%-47.9%]), India (45.1% [95% CrI, 43.7%-46.6%] vs 30.4% [95% CrI, 29.2%-31.6%]), Indonesia (35.9% [95% CrI, 27.7%-44.0%] vs 17.9% [95% CrI, 11.7%-24.7%]), and Nepal (74.3% [95% CrI, 70.3%-78.2%] vs 56.6% [95% CrI, 52.2%-60.7%]). There was greater uncertainty about whether the differences observed for Cambodia (47.4% [95% CrI, 38.1%-56.6%] vs 32.6% [95% CrI, 25.8%-39.9%]), Laos (81.0% [95% CrI, 71.1%-89.7%] vs 75.0% [95% CrI, 64.8%-84.1%]), Pakistan (42.2% [95% CrI, 39.1%-45.0%] vs 37.4% [95% CrI, 34.4%-40.3%]), and Vietnam (36.2% [95% CrI, 24.5%-49.0%] vs 21.5% [95% CrI, 12.0%-32.9%]) were due to chance variation.

Meropenem coverage was higher than aminopenicillin-gentamicin coverage in Cambodia (90.6% [95% CrI, 86.2%-94.4%] vs 47.4% [95% CrI, 38.1%-56.6%]), China (76.5% [95% CrI, 71.8%-80.9%] vs 60.6% [95% CrI, 54.2%-67.5%]), India (64.0% [95% CrI, 62.6%-65.4%] vs 45.1% [95% CrI, 43.7%-46.6%]), Indonesia (88.8% [95% CrI, 83.2%-93.6%] vs 35.9% [95% CrI, 27.7%-44.0%]), Pakistan (88.1% [95% CrI, 85.6%-90.3%] vs 42.2% [95% CrI, 39.1%-45.0%]), and Vietnam (84.1% [95% CrI, 73.2%-92.6%] vs 36.2% [95% CrI, 24.5%-49.0%]) on the basis of nonoverlapping 95% CrIs. The largest percentage differences in coverage were observed in Indonesia (52.9%), Pakistan (45.9%), and Cambodia (43.2%); the smallest was in China (15.9%). For meropenem and third-generation cephalosporins, the percentage difference was largest for Indonesia (70.9%), Vietnam (62.6%), and Cambodia (58.0%). Of note, for Laos and Nepal, imprecision around estimated meropenem coverage, which was comparable with that of aminopenicillin-gentamicin with overlapping 95% CrIs, was largely because of low proportions of isolates (62.5% [40 of 64] for Laos and 60.3% [295 of 489] for Nepal) contributing to the meropenem susceptibility parameter.

## Discussion

We estimated the coverage offered by 3 antibiotic regimens—aminopenicillin-gentamicin (WHO-recommended first-line regimen), third-generation cephalosporins (WHO-recommended second-line regimen), and meropenem—in Asian countries for the empirical treatment of neonatal sepsis caused by 7 specified bacteria. The coverage estimates were based on a systematic review of recent studies reporting on the relative incidence of common bacteria and their resistance.

In general, coverage estimates supported the identification of better-performing or worse-performing regimens for most countries. Coverage offered by aminopenicillin-gentamicin (WHO-recommended first-line regimen) was less than 50% for Cambodia, India, Indonesia, Pakistan, and Vietnam and less than 75% for China and Nepal. Even lower coverage was offered by the WHO-recommended second-line third-generation cephalosporin monotherapy regimen: below 50% in all represented countries except Laos (75.0%) and Nepal (56.6%). Meropenem coverage was generally highest and was greater than 80% in Cambodia, Indonesia, Pakistan, and Vietnam, but lower than 80% in China, Laos, and Nepal and as low as 64.0% in India. Considerable between-country differences were observed for all 3 regimens, even for countries bordering each other, such as Cambodia, Laos, Thailand, and Vietnam.

Coverage estimates are clinically highly relevant for the development of local and national empirical treatment guidelines, incorporating both the relative incidence of bacteria and their susceptibility.^9^ This concept has not, to our knowledge, been previously applied to neonatal sepsis in LMICs. Instead, reports have focused on susceptibility for individual pathogen-drug combinations, an approach that does not directly incorporate the spectrum of causative bacteria.^4,6,7^

One important question is whether global setting-independent recommendations for empirical neonatal sepsis treatment can be supported in an era of changing and highly variable epidemiology. In some settings, difficult-to-treat pathogens and multidrug-resistant isolates now contribute considerably to neonatal sepsis.^3^ Stratified guidance moving between recommended regimens according to microbiology and coverage by patient-level factors (eg, presence of certain underlying conditions or timing of sepsis onset) or setting, may be a solution. One challenge will be the lack of defined coverage thresholds to move between regimens.^21^ Given sufficiently large data sets, coverage estimates could help inform such shifting by supporting inferences about true differences between regimens.

### Limitations

This study has some limitations. Our coverage estimates were based on data from predominantly university or teaching hospitals. Infants with complex medical issues and those at higher risk of nosocomial bloodstream infections may, therefore, be overrepresented. At the same time, microbiology data from infants managed in district hospitals are lacking precluding confirmation that presented coverage estimates are applicable to them as well. Clinicians applying WHO recommendations to infants with nosocomial infection or those managed in tertiary hospitals would, on the basis of our observations, need to consider alternatives for this population.

We chose to estimate coverage according to the pathogens frequently reported across included studies, which are likely to be associated with severe neonatal sepsis and the so-called ESKAPE organisms (ie, *Enterococcus faecium*, *S aureus*, *Klebsiella pneumoniae*, *Acinetobacter baumannii*, and *Pseudomonas aeruginosa*), which are known to be problematic in terms of emerging antimicrobial resistance.^22^ Inclusion of other pathogens would be expected to have a variable influence on the expected coverage of considered antibiotics, leading to either higher or lower estimates. This may be particularly important in individual hospitals with ongoing outbreaks where a single bacterial strain is dominant. In such situations, regional coverage estimates may not be applicable.

Coverage estimation requires a number of assumptions to be made when calculating the susceptibility parameters, such as the incorporation of intrinsic resistance, extrapolations from susceptibility testing for 1 representative of an antibiotic class to other members of this class, and the interpretation of multiple testing for 1 antibiotic class. We based our calculations of regimen susceptibility on EUCAST algorithms and, whenever possible, used susceptibility testing information for the specific drug of interest.^19^ Importantly, however, all included studies used versions of Clinical and Laboratory Standards Institute interpretive criteria,^23^ which may diverge from EUCAST in terms of both break points and assumptions about intrinsic resistance. Debate about the merits and challenges of switching from Clinical and Laboratory Standards Institute to EUCAST and about the implications of such a transition for interpretation of routine data in the context of surveillance is ongoing.^23,24^

To support coverage estimation, it is important that the microbiological data used are collected in equivalent ways. However, the data used for this analysis may have been subject to various random or systematic errors that could bias the coverage estimates. Possible sources of error include duplicate isolates, contaminants, nonstandardized susceptibility testing, combining data from different patient populations (children and adults), and reflex susceptibility testing based on resistance identified in a first-line testing panel.^25^ These requirements have important implications for global surveillance initiatives, such as the Global Antimicrobial Resistance Surveillance System,^26^ if data collected are to be used at the interface between surveillance and clinical practice.

## Conclusions

Recently, machine learning approaches and more elaborate multivariable Bayesian models using clinical and demographic information combined with microbiological data have been proposed as optimizing the selection of empirical antibiotic treatment for sepsis.^27,28^ Although these models may help in selecting patient-adapted regimens, the approach used in our study only requires estimates of pathogen incidence and susceptibility and could already substantially improve clinical decision-making based on routine microbiological data alone, provided that the data used to produce these estimates are of sufficient quality. Our analysis indicates that the recommendation for third-generation cephalosporin monotherapy as a second-line regimen may no longer be valid for many infants receiving treatment for neonatal sepsis in several Asian countries. Our findings could explain the high reported empirical meropenem use in this population in Asia.^14,29^ Evaluation of potential alternatives will be essential to reducing consumption of last-resort antibiotics for the empirical treatment of neonatal sepsis in settings with a high prevalence of antimicrobial resistance.

## Notes

Supplement. **eAppendix.** Supplemental Methods **eFigure 1. **Illustration of Decision Tree for Estimating Coverage From Weighted Incidence Syndromic Combination Antibiograms for Three Antibiotic Regimens of Interest **eFigure 2. **Flow Chart: Systematic Review of the Literature
eReferences.
**eTable 1. **Description of Included Publications **eTable 2.** Information on Sample Processing Provided in Included Publications **eTable 3. **Relative Incidence of Bacteria in Included Studies
