# Supplementary material for: Evaluation of the Coverage of 3 Antibiotic Regimens for Neonatal Sepsis in the Hospital Setting Across Asian Countries
Source: JAMA Netw Open. 2020 Feb 12;3(2):e1921124. doi: 10.1001/jamanetworkopen.2019.21124 (PMC11893050; doi:10.1001/jamanetworkopen.2019.21124)
Supplement: Supplement. — eAppendix. Supplemental Methods eFigure 1. Illustration of Decision Tree for Estimating Coverage From Weighted Incidence Syndromic Combination Antibiograms for Three Antibiotic Regimens of Interest eFigure 2. Flow Chart: Systematic Review of the Literature eReferences. eTable 1. Description of Included Publications eTable 2. Information on Sample Processing Provided in Included Publications eTable 3. Relative Incidence of Bacteria in Included Studies [file jamanetwopen-e1921124-s001.pdf]

Supplementary Online Content

Bielicki JA, Sharland M, Heath PT, et al. Evaluation of the coverage of 3 antibiotic regimens for neonatal sepsis in the hospital setting across Asian countries. *JAMA Netw Open*. 2020:3(2):e1921124. doi:10.1001.jamanetworkopen.2019.21124

eAppendix. Supplemental Methods
eFigure 1. Illustration of Decision Tree for Estimating Coverage From Weighted Incidence Syndromic Combination Antibiograms for Three Antibiotic Regimens of Interest
eFigure 2. Flow Chart: Systematic Review of the Literature
eReferences.
eTable 1. Description of Included Publications
eTable 2. Information on Sample Processing Provided in Included Publications
eTable 3. Relative Incidence of Bacteria in Included Studies

This supplementary material has been provided by the authors to give readers additional information about their work.

© 2020 Bielicki JA et al. *JAMA Network Open.*

eAppendix. Supplemental Methods

Search strategy for systematic literature review

Ovid MEDLINE® 1946 to April 25 2019

1	exp SEPSIS/ or exp NEONATAL SEPSIS/
2	exp BACTEREMIA
3	bacter?emia.mp. [mp=title, abstract, original title, name of substance word, subject heading word, keyword heading word, protocol supplementary concept word, rare disease supplementary concept word, unique identifier, synonyms]
4	(blood?stream adj3 infect*).mp. [mp=title, abstract, original title, name of substance word, subject heading word, keyword heading word, protocol supplementary concept word, rare disease supplementary concept word, unique identifier, synonyms]
5	(blood adj2 culture adj2 (positive* or isolat*)).mp. [mp=title, abstract, original title, name of substance word, subject heading word, keyword heading word, protocol supplementary concept word, rare disease supplementary concept word, unique identifier, synonyms]
6	1 or 2 or 3 or 4 or 5
7	((anti?biotic* or anti?infect* or anti?microb*) adj2 (resist* or suscep* or sensitive*)).mp. [mp=title, abstract, original title, name of substance word, subject heading word, keyword heading word, protocol supplementary concept word, rare disease supplementary concept word, unique identifier, synonyms]
8	exp Drug Resistance, Microbial/
9	7 or 8
10	exp infant/ or exp infant, newborn/
11	(infant* or neonat* or new?born).mp. [mp=title, abstract, original title, name of substance word, subject heading word, keyword heading word, protocol supplementary concept word, rare disease supplementary concept word, unique identifier, synonyms]
12	10 or 11
13	6 and 9 and 12
14	Exp ASIA/
15	13 and 14
16	Limit 15 to yr=”2014-Current”

Embase 1974 to 2019 Week 16

1	exp bacteremia/
2	exp sepsis/ or newborn sepsis/
3	bacter?emia.mp. [mp=title, abstract, heading word, drug trade name, original title, device manufacturer, drug manufacturer, device trade name, keyword, floating subheading word, candidate term word]
4	(blood?stream adj2 infect*).mp. [mp=title, abstract, heading word, drug trade name, original title, device manufacturer, drug manufacturer, device trade name, keyword, floating subheading word, candidate term word]
5	(blood adj2 culture adj2 (positive* or isolate*)).mp. [mp=title, abstract, heading word, drug trade name, original title, device manufacturer, drug manufacturer, device trade name, keyword, floating subheading word, candidate term word]
6	1 or 2 or 3 or 4 or 5
7	((anti?biotic* or anti?infect* or anti?microb*) adj2 (resist* or suscep* or sensitiv*)).mp. [mp=title, abstract, heading word, drug trade name, original title, device manufacturer, drug manufacturer, device trade name, keyword, floating subheading word, candidate term word]
8	exp antibiotic resistance/
9	7 or 8
10	infant/
11	newborn/
12	(infant or new?born or neonat*).mp. [mp=title, abstract, heading word, drug trade name, original title, device manufacturer, drug manufacturer, device trade name, keyword, floating subheading word, candidate term word]
13	10 or 11 or 12
14	6 and 9 and 13
15	14
16	14 and 15

© 2020 Bielicki JA et al. *JAMA Network Open.*

17 limit 16 to yr="2014-Current"

Systematic review of the literature: selection of publications
Studies were eligible for inclusion if they examined blood culture isolates and (i) provided information specific to newborns up to 28 days of age or infants managed on neonatal units, (ii) reported on the relative incidence of different bacteria at species or genus level during the indicated surveillance period and (iii) included data on antimicrobial resistance for at least one bacterial species or genus. Publications reporting on isolates from sources other than blood, and those from which data for neonatal blood cultures (e.g. reporting pooled data across age groups) could not be extracted were excluded. Equally studies focusing on single organisms from which the relative incidence of other bacteria could not be obtained were excluded. Further we excluded studies presenting only aggregate data by region or internationally.

After exclusion of duplicates, titles or abstracts of retrieved studies were reviewed by one author (JB) to identify those meeting inclusion criteria. A random subset of retrieved studies was reviewed by a second author (MS) to ensure consistency in selection based on the pre-specified inclusion and exclusion criteria with no disagreements.

Selected publications were primarily used to inform parameter estimation for calculating coverage. Additional extracted data included contextual information (namely the year of publication, the country from where the data originated, the surveillance/reporting period, and the number and type of hospitals surveyed), and whether studies reported on blood culture isolates from community-acquired infections, hospital-acquired infections or both. Early onset of neonatal sepsis defined as infection occurring in the first 3 days of life was considered a community-acquired infection. We also extracted information on approaches to species identification, susceptibility testing and evaluation of testing results, if provided. Species identification and susceptibility testing results were recorded as reported. As the study was focused on the reporting of routine microbiological or surveillance data, we did not undertake a formal grading of the quality of the studies or an evaluation of the appropriateness of microbiological approaches.

Assumptions for determining susceptibility of pathogens to pre-specified regimens
- Aminopenicillin susceptibility was based on either ampicillin or amoxicillin susceptibility testing results, whichever was available.
- Gentamicin susceptibility was based on results for gentamicin rather than other aminoglycosides whenever possible, because susceptibility to gentamicin cannot be reliably inferred from results for other aminoglycosides. If no gentamicin susceptibility data were provided, data from other aminogylcosides (mostly amikacin) were used.
- Third-generation cephalosporin susceptibility was based on either cefotaxime or ceftriaxone, whichever was available.
- Meropenem susceptibility was based on results for meropenem rather than other carbapenems whenever possible, because susceptibility to meropenem cannot be reliably inferred from results for other carbapenems. If no meropenem susceptibility data were provided, data from other carbapenems (mostly imipenem) were used.
- For *Staphylococcus aureus*, third-generation cephalosporin and meropenem susceptibility was derived from information on methicillin resistance, as these antibiotics are not generally specifically tested for *S. aureus*.
- For the combined regimen (i), the one with the higher susceptibility was taken to reflect overall susceptibility. For example, if *Escherichia coli* in a specific country exhibited 20% ampicillin susceptibility and 70% gentamicin susceptibility, susceptibility to aminopenicillin plus gentamicin for *E. coli* was assumed to be 70%.

Technical appendix on calculation of the weighted-incidence syndromic combination antibiogram (WISCA)
In the WISCA decision tree, the first square node represents the clinical decision to start empiric antibiotic therapy and the regimen choices. Subsequent circular nodes and branches describe chance events, which are the range of relevant bacteria causing neonatal sepsis, their relative incidence and the percentages of each pathogen susceptible to each antibiotic regimen. Combining the probabilities along the regimen tree branches provides an estimate of coverage for each regimen.

A difficulty in adopting a Bayesian perspective is the specification of the prior distributions for the parameters. The value of the relative incidence and pathogen–regimen susceptibility parameters for each regimen were therefore defined as probability distributions that reflected the uncertainty in their value. Given that susceptibility percentages are simple proportions, we selected a binomial distribution to describe our prior belief defined using the conjugate Beta distribution. This approach results in the posterior also being a Beta distribution. The relative incidence data were assumed to be drawn from a multinomial distribution with nine possible outcomes. The prior was accordingly modeled as a Dirichlet (1,1,1,…,1) distribution. This is the continuous equivalent to the discrete multinomial distribution, and is the generalisation of the Beta distribution to situations described by more than two categories.

© 2020 Bielicki JA et al. *JAMA Network Open.*

In the absence of any strong prior beliefs, a common solution is to use a “non-informative” uniform prior. Doing this means that the posterior distribution is largely determined by the observed data. Using the Dirichlet distribution as the prior, for example, results in the posterior taking the form Dirichlet (1+n1, 1+n2,. . ., 1+n9). Equally, in most cases, when there were no strong prior beliefs about pathogen-regimen susceptibility, the non-informative prior beta(1,1) was used.

Adopting a Bayesian perspective allows the use of informative priors for the situation in which a pathogen has intrinsic resistance or is assumed to be fully susceptible. For these, we chose a pragmatic posterior Beta distribution, chosen to have an appropriate standard deviation. For example, susceptibility for a pathogen with intrinsic resistance was specified as a Beta(1,9999), which has a standard deviation of 0.01%. Sampling from this distribution only gives pathogen resistance below 99.9% in 1 in 20000 draws.

The calculation of the 95% credible interval describing the precision of coverage estimates requires Monte Carlo simulation, which involves running a large number of experiments (in our case 1000) and combining their results. In each experiment, parameter values for the parameters of interest (relative incidence and pathogen-regimen susceptibility) are randomly drawn from their specified distributions. The values of each parameter are then combined to derive a coverage estimate. Together, the individual coverage estimates from all the experiments give the posterior distribution for the coverage parameter. The 95% “uncertainty” interval, or 95% credible interval, is then calculated as the interval between 2.5% and 97% percentile of this distribution.

Analytical steps for basic WISCA coverage estimation using a Bayesian decision tree model.

1. Identify the total number of isolates contributing to the infection syndrome of interest for a given setting and period.
2. Select from 1. clinically relevant bacteria contributing to the infection syndrome and with data available to define model parameters.
3. Specify assumptions used for determining susceptibility to the regimen, including extrapolation from standard bug-drug susceptibility testing, definitions of intrinsic resistance and, when relevant, intrinsic susceptibility (corresponding to unusual resistance phenotypes)
4. For the bacteria specified in 2. identify the number of isolates contributed by each (to determine relative frequency = first circular node and branches) and the number of isolates tested for and susceptible to the regimen of interest (second circular node and branches).
5. Select appropriate informative priors for bacteria with intrinsic resistance or expected susceptibility as set out in 3.
6. Select non-informative priors for relative bacterial incidence and susceptibility with the exceptions as outlined in 5.
7. Use appropriate probability distributions to reflect uncertainty in the relative frequency of bacteria (multinomial, Dirichlet distribution) and susceptibility to the regimen (binomial, Beta distribution).
8. Model coverage by running a Monte Carlo simulation with n experiments sampling parameter values for relative bacterial frequency and regimen susceptibility from their specified distributions.
9. Combine estimates from n experiments to calculate coverage estimates with their 2.5% and 97% percentiles, corresponding to the 95% uncertainty or credible interval.
10. Repeat this process for each regimen of interest, noting that for comparisons within a given setting the bacteria included in the WISCA should stay the same (meaning that number of isolates contributed by each will be the same), but that the number tested and susceptible will vary by regimen.

© 2020 Bielicki JA et al. *JAMA Network Open.*

eFigure 1. Illustration of Decision Tree for Estimating Coverage From Weighted Incidence Syndromic Combination Antibiograms for Three Antibiotic Regimens of Interest

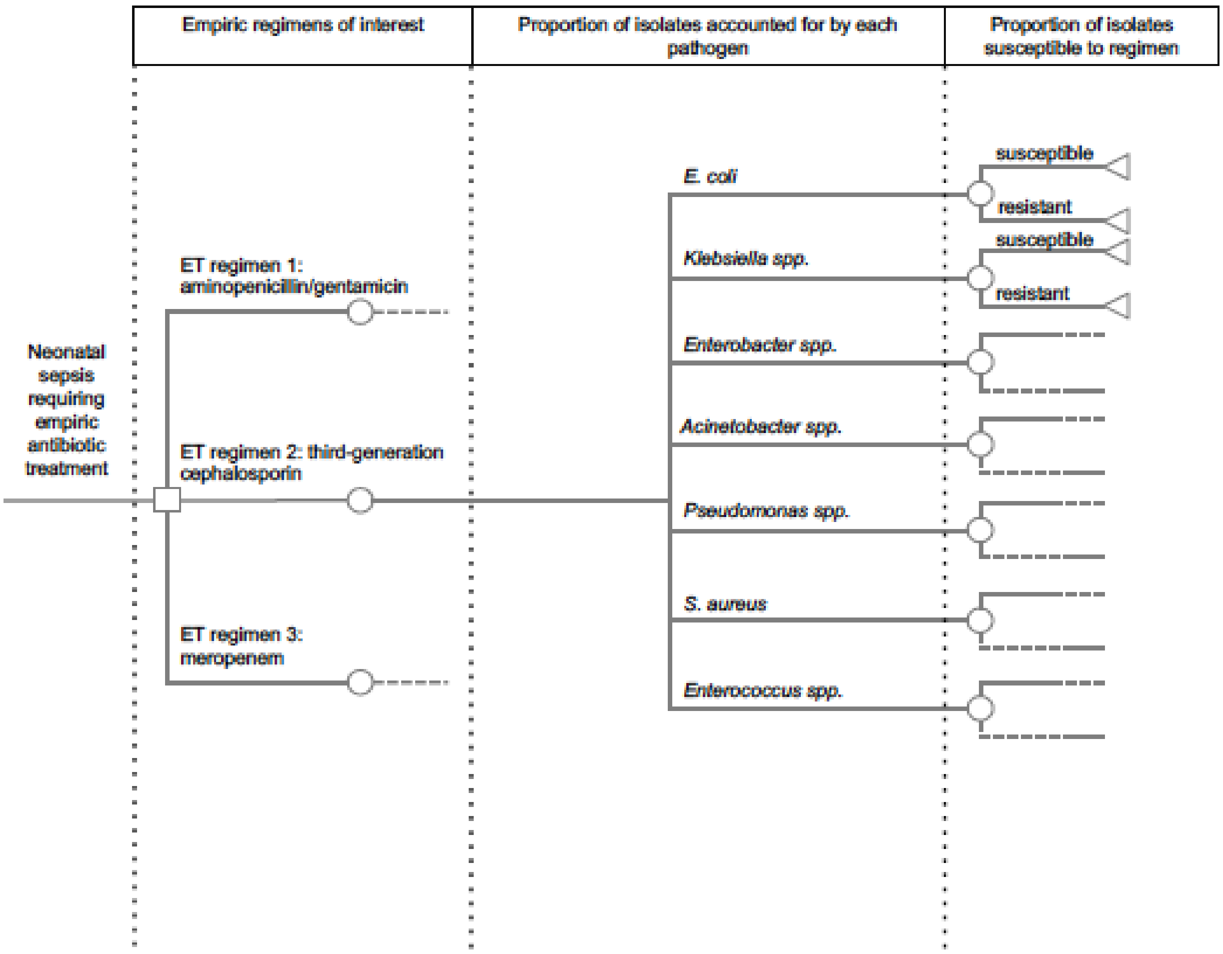

ET: empiric therapy. Square node: clinical decision to treat; circular node: chance event (causal bacteria and their regimen susceptibility). The decision tree is shown for illustration only, and dashed lines indicate where the decision tree has been left incomplete. All branches are included in the WISCA calculations to estimate coverage.

© 2020 Bielicki JA et al. *JAMA Network Open.*

eFigure 2. Flow Chart: Systematic Review of the Literature

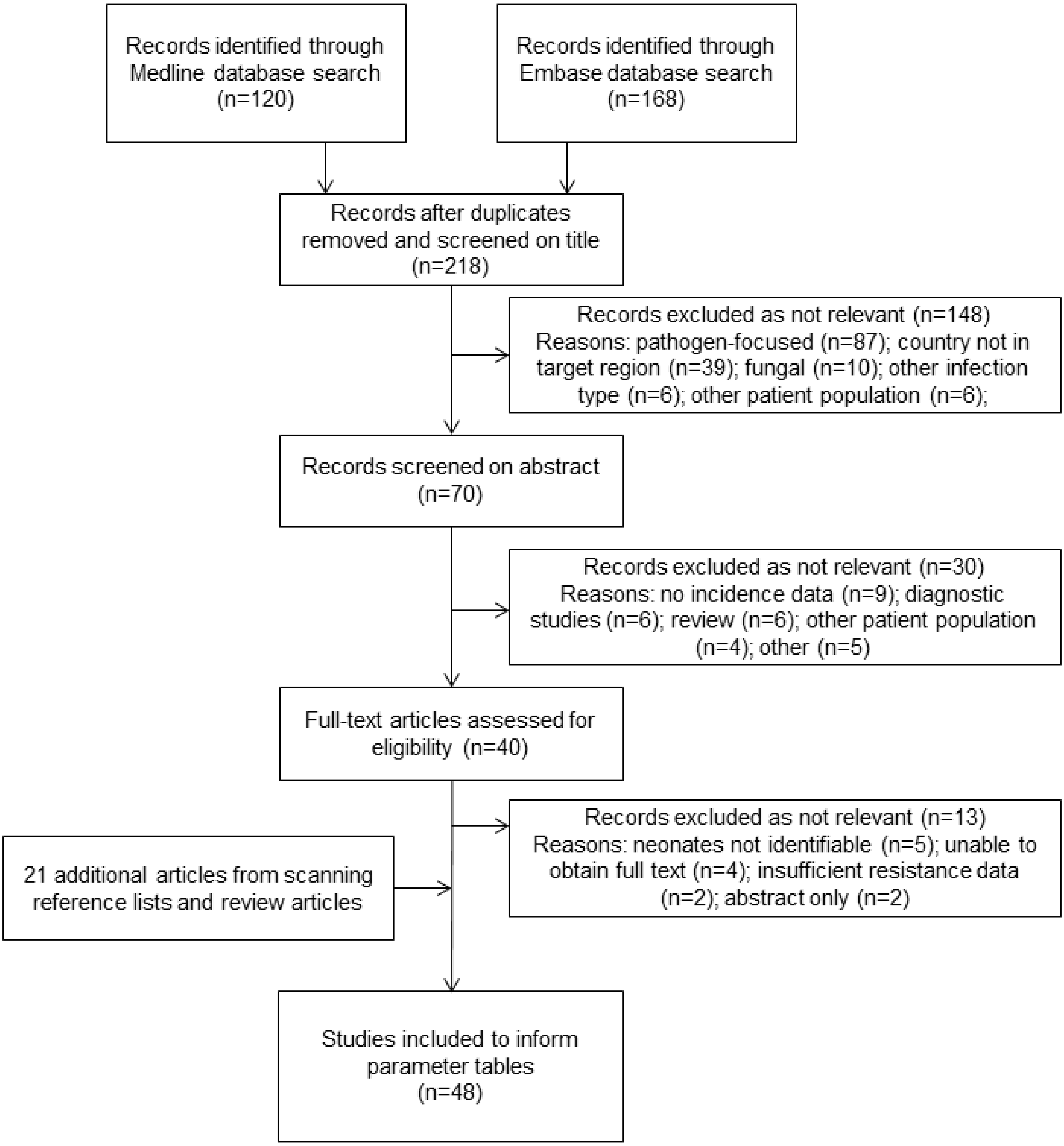

© 2020 Bielicki JA et al. *JAMA Network Open.*

eReferences: Reference list for included publications

1. Abu NA, Nor FM, Mohamad M, et al. Community-acquired Bacteremia in paediatrics: Epidemiology, aetiology and patterns of antimicrobial resistance in a tertiary care centre, Malaysia. *Medical Journal of Malaysia* 2016; **71**(3): 117-21.
2. Adhikari N, Shah PK, Acharya G, Vaidya KM. Bacteriological profile and associated risk factors of neonatal sepsis in Paropakar Maternity and Women's Hospital Thapathali, Kathmandu. *Nepal Medical College journal : NMCJ* 2014; **16**(2-4): 161-4.
3. Agarwal A, Bhat S. Clinico-microbiological study of neonatal sepsis. *Journal of International Medicine and Dentistry* 2015; **2**(1): 22-9.
4. Ambade VN, Kolpe D, Tumram N, Meshram S, Pawar M, Kukde H. Characteristic Features of Hanging: A Study in Rural District of Central India. *Journal of forensic sciences* 2015; **60**(5): 1216-23.
5. Amin AJ, Malam PP, Asari PD, Patel UR, Behl AB. Sensitivity and resistance pattern of antimicrobial agents used in cases of neonatal sepsis at a tertiary care centre in western India. *International Journal of Pharmaceutical Sciences and Research* 2016; **7**(7): 3060-7.
6. Anderson M, Luangxay K, Sisouk K, et al. Epidemiology of bacteremia in young hospitalized infants in vientiane, laos, 2000-2011. *Journal of Tropical Pediatrics* 2014; **60**(1): 10-6.
7. Chapagain RH, Acharya R, Shrestha N, Giri BR, Bagale BB, Kayastha M. Bacteriological Profile of Neonatal Sepsis in Neonatal Intermediate Care Unit of Central Paediatric Referral Hospital in Nepal. *Journal of Nepal Health Research Council* 2015; **13**(31): 205-8.
8. Dalal P, Gathwala G, Gupta M, Singh J. Bacteriological profile and antimicrobial sensitivity pattern in neonatal sepsis: a study from North India. *2017* 2017; **5**(4): 5.
9. Dhanalakshmi V, Sivakumar ES. Comparative Study in Early Neonates with Septicemia by Blood Culture, Staining Techniques and C - Reactive Protein (CRP). *Journal of clinical and diagnostic research : JCDR* 2015; **9**(3): Dc12-5.
10. Dhaneria M, Jain S, Singh P, Mathur A, Lundborg CS, Pathak A. Incidence and Determinants of Health Care-Associated Blood Stream Infection at a Neonatal Intensive Care Unit in Ujjain, India: A Prospective Cohort Study. *Diseases* 2018; **6**(1).
11. Dong H, Cao H, Zheng H. Pathogenic bacteria distributions and drug resistance analysis in 96 cases of neonatal sepsis. *BMC Pediatrics* 2017; **17**(1): 44.
12. Fox-Lewis A, Takata J, Miliya T, et al. Antimicrobial resistance in invasive bacterial infections in hospitalized children, Cambodia, 2007-2016. *Emerging Infectious Diseases* 2018; **24**(5): 841-51.
13. Gupta M, Chaudhary U. Bacteriologic profile and antibiogram of paediatric blood cultures in a tertiary care centre. *International Journal of Pharma and Bio Sciences* 2015; **6**(2): B341-B6.
14. Ingale HD, Kongre VA, Bharadwaj RS. A study of infections in neonatal intensive care unit at a tertiary care hospital. *2017* 2017; **4**(4): 8.
15. Investigators of the Delhi Neonatal Infection Study c. Characterisation and antimicrobial resistance of sepsis pathogens in neonates born in tertiary care centres in Delhi, India: a cohort study. *Lancet Glob Health* 2016; **4**(10): e752-60.
16. Jajoo M, Manchanda V, Chaurasia S, et al. Alarming rates of antimicrobial resistance and fungal sepsis in outborn neonates in North India. *PLoS ONE* 2018; **13 (6)** (e0180705).
17. Javali NS, Banu N, Indi SM. Incidence and Microbiological Profile of Late Onset Neonatal Sepsis in Preterm and Low Birth Weight Neonates, NICU, RIMS, Raichur. *J of Evidence Based Med & Hlthcare* 2014; **1**(16): 2036-48.
18. Jiang Y, Kuang L, Wang H, Li L, Zhou W, Li M. The clinical characteristics of neonatal sepsis infection in Southwest China. *Internal Medicine* 2016; **55**(6): 597-603.
19. Kamble R, Ovhal R. Bacteriological profile of neonatal septicemia. *International Journal of Current Microbiology and Applied Sciences* 2015; **4**(2): 172-82.
20. Kanodia P, Yadav SK, Sigh RR, Bhatta NK. Bacteriological Profile of Blood Culture Positive Sepsis in Newborn at BPKIHS, Dharan Nepal. *Journal of College of Medical Sciences - Nepal* 2017; **13**(1): 193-6.
21. Khanal R, Manandhar S, Acharya GP. Bacteriological profile of neonatal sepsis in a tertiary level hospital of Nepal. *Journal of Nepal Paediatric Society* 2014; **34**(3): 175-80.
22. Li X, Ding X, Shi P, et al. Clinical features and antimicrobial susceptibility profiles of culture-proven neonatal sepsis in a tertiary children's hospital, 2013 to 2017. *Medicine* 2019; **98**(12): e14686.
23. Lu Q, Zhou M, Tu Y, Yao Y, Yu J, Cheng S. Pathogen and antimicrobial resistance profiles of culture-proven neonatal sepsis in Southwest China, 1990-2014. *Journal of Paediatrics and Child Health* 2016; **52**(10): 939-43.
24. Madavi D, Aziz F, Agrawal G. Clinica-Bacteriological Profile and Antibiotic Sensitivity Pattern of Neonatal Septicaemia - A Prospective Observational Study. *International Journal of Current Research and Review* 2015; **7**(5): 13-20.
25. Mahmood T, Javed N, Subhani H, Ali FA. Bacteria isolated from Blood Cultures of Septicemic children at a teaching Unit. *Pakistan Journal of Medical and Health Sciences* 2016; **10**(4): 1129-31.

© 2020 Bielicki JA et al. *JAMA Network Open.*

26. Marwah P, Chawla D, Chander J, Guglani V, Marwah A. Bacteriological profile of neonatal sepsis in a tertiary-care hospital of Northern India. *Indian pediatrics* 2015; **52**(2): 158-9.
27. Mehta A.M., Navinchandra M. K., Tukaram K.P. Microbial Profile of Neonatal septicaemia in a tertiary care hospital of Bhopal. *International Journal of Biomedical and Advance Research* 2014; **5**(10): 499-501.
28. Muley VA, Ghadage DP, Bhore AV. Bacteriological profile of neonatal septicemia in a tertiary care hospital from Western India. *Journal of Global Infectious Diseases* 2015; **7**(2): 75-7.
29. Mustafa M., Ahmed S.L. Bacteriological profile and antibiotic susceptiblity patterns in neonatal septicemia in view of emerging drug resistance. *Journal of Medical and Allied Sciences* 2014; **4**(1): 2-8.
30. Nayak S, Rai R, Kumar V, Sanjeev H, Pai A, Ganesh H. Distribution of microorganisms in neonatal sepsis and antimicrobial susceptibility patterns in a tertiary care hospital. *Archives of Medicine and Health Sciences* 2014; **2**(2): 136-9.
31. Pandita N, Wasim S, Bhat NK, Chandra V, Kakati B. Identification of the bacterial isolates in neonatal septicaemia and their antimicrobial susceptibility in a tertiary care hospital in Uttarakhand, India: a retrospective study. *2016* 2016; **3**(1): 6.
32. Panigrahi P, Chandel DS, Hansen NI, et al. Neonatal sepsis in rural India: Timing, microbiology and antibiotic resistance in a population-based prospective study in the community setting. *Journal of Perinatology* 2017; **37**(8): 911-21.
33. Patel D, Nimbalkar A, Sethi A, Kungwani A, Nimbalkar S. Blood culture isolates in neonatal sepsis and their sensitivity in Anand District of India. *Indian journal of pediatrics* 2014; **81**(8): 785-90.
34. Pavan Kumar DV, Mohan J, Rakesh PS, Prasad J, Joseph L. Bacteriological profile of neonatal sepsis in a secondary care hospital in rural Tamil Nadu, Southern India. *Journal of family medicine and primary care* 2017; **6**(4): 735-8.
35. Pokhrel B, Koirala T, Shah G, Joshi S, Baral P. Bacteriological profile and antibiotic susceptibility of neonatal sepsis in neonatal intensive care unit of a tertiary hospital in Nepal. *BMC Pediatrics* 2018; **18:** 208.
36. Ponugoti ML, Venkatakrishna M, Jithendra K, Reddy PS. Incidence of Early Onset Septicemia, Isolation and Resistant Patterns of Causative Organisms: A Study in a Tertiary Care Hospital A.P. *Journal of Medical Science and Clinical Research* 2015; **3**(2): 4227-32.
37. Roy MP, Bhatt M, Maurya V, Arya S, Gaind R, Chellani HK. Changing trend in bacterial etiology and antibiotic resistance in sepsis of intramural neonates at a tertiary care hospital. *Journal of postgraduate medicine* 2017; **63**(3): 162-8.
38. Sarangi KK, Pattnaik D, Mishra SN, Nayak MK, Jena J. Bacteriological profile and antibiogram of blood culture isolates done by automated culture and sensitivity method in a neonatal intensive care unit in a tertiary care hospital in Odisha, India. *2017* 2017; **2**(4): 6.
39. Sari IP, Nuryastuti T, Wahyono D. The study of multidrug-resistance in neonatal intensive care unit at the central java hospital. *Asian Journal of Pharmaceutical and Clinical Research* 2017; **10**(Special Issue may): 80-4.
40. Singh HK, Sharja P, Onkar K. Bacteriological profile of neonatal sepsis in neonatal intensive care unit (NICU) in a tertiary care hospital: prevalent bugs and their susceptibility patterns. *European Journal of Pharmaceutical and Medical Research* 2016; **3**(3): 241-5.
41. Thakur S, Thakur K, Sood A, Chaudhary S. Bacteriological profile and antibiotic sensitivity pattern of neonatal septicaemia in a rural tertiary care hospital in North India. *Indian Journal of Medical Microbiology* 2016; **34**(1): 67-71.
42. Ting YT, Lu CY, Shao PL, et al. Epidemiology of community-acquired bacteremia among infants in a medical center in Taiwan, 2002-2011. *Journal of microbiology, immunology, and infection = Wei mian yu gan ran za zhi* 2015; **48**(4): 413-8.
43. Tran HT, Doyle LW, Lee KJ, Dang NM, Graham SM. A high burden of late-onset sepsis among newborns admitted to the largest neonatal unit in central Vietnam. *Journal of Perinatology* 2015; **35**(10): 846-51.
44. Tudu NK, Dey R, Bhattacharya I, Roy S, Dey JB. A pilot study on bacterial profile of neonatal sepsis in a tertiary care hospital serving rural population. *Journal of Evolution of Medical and Dental Sciences* 2014; **3**(23): 6378-81.
45. Ullah O, Khan A, Ambreen A, et al. Antibiotic Sensitivity pattern of Bacterial Isolates of Neonatal Septicemia in Peshawar, Pakistan. *Arch Iran Med* 2016; **19**(12): 866-9.
46. Venkatnarayan K, Bej PK, Thapar RK. Neonatal Sepsis: A Profile of a Changing Spectrum. *J Nepal Paediatr Soc* 2014; **34**(3): 207-14.
47. Wang S, Chen S, Feng W, et al. Clinical characteristics of nosocomial bloodstream infections in neonates in two hospitals, China. *Journal of Tropical Pediatrics* 2018; **64**(3): 231-6.
48. Yadav NS, Sharma S, Chaudhary DK, et al. Bacteriological profile of neonatal sepsis and antibiotic susceptibility pattern of isolates admitted at Kanti Children's Hospital, Kathmandu, Nepal. *BMC research notes* 2018; **11**(1): 301.

© 2020 Bielicki JA et al. *JAMA Network Open.*

eTable 1. Description of Included Publications

Publication year, First author, Journal			Country, City/Town		N hospitals, type		Observation period start and end*		Infections surveyed
2014	Adhikari	Nepal Medical College Journal	Nepal	Thapathali	1	Maternity	01Aug11	31Mar12	Sepsis with positive BC
	Anderson	Journal of Tropical Pediatrics	Laos	Vientiane	1	U/T	01Feb00	01Sep11	Sepsis with positive BC
	Javali	Journal of Evidence Based Medicine & Healthcare	India	Raichur	1	NT/D	01Jun13	30Jul13	LONS with positive BC
	Khanal	Journal of Nepal Paediatric Society	Nepal	Kathmandu	1	Maternity	01Dec10	31Mar11	Sepsis with positive BC
	Mehta	International Journal of Biomedical And Advance Research	India	Bhanpur	1	U/T	01Jul12	31Dec13	Sepsis with positive BC
	Mustafa	Journal of Medical and Allied Sciences	India	Hyderabad	1	U/T	Unknown (1 year)		Sepsis with positive BC
	Nayak	Archives of Medicine and Health Sciences	India	Deralakatte	1	U/T	01Jun11	31May12	Sepsis with positive BC
	Patel	The Indian Journal of Pediatrics	India	Karamsad	1	NT/D	01Nov07	31Oct11	Bacteraemia
	Tudu	Journal of Evoluation of Medical and Dental Science	India	Kenduadihi	1	U/T	01Jun13	31Aug13	Sepsis with positive BC
	Venkatna rayan	Journal of Nepal Paediatric Society	India	Pune	1	U/T	01Jan11	01Jul12	Sepsis with positive BC
2015	Agarwal	Journal of International Medicine and Dentistry	India	Mangalore	1	U/T	01Feb14	31Jul14	Sepsis with positive BC
	Ambade	Journal of Medical Science and Clinical Research	India	Dhule	1	U/T	01Aug12	31Jul14	Sepsis with positive BC
	Chapagai n	Journal of the Nepalese Health Research Council	Nepal	Kathmandu	1	Paediatric	01Aug14	01Aug15	Sepsis with positive BC
	Dhanalak shmi	Journal of Clinical and Diagnostic Research	India	Madurai	1	U/T	01Dec13	30Sep2014	Sepsis with positive BC
	Gupta	International Journal of Pharma and Bio Sciences	India	Rohtak	1	NT/D	Unknown (1year)		Bacteraemia
	Kamble	International Journal of Current	India	Ambajogai	1	U/T	01Jun08	21Dec10	Sepsis with positive BC

© 2020 Bielicki JA et al. *JAMA Network Open.*

		Microbiology and Applied Sciences							
	Madavi	International Journal of Current Research and Review	India	Nagpur	1	U/T	01Aug11	01Sep13	Sepsis with positive BC
	Marwah	Indian Pediatrics	India	Chandigarh	1	U/T	01Jan08	31Dec12	Bacteraemia
	Muley	Journal of Global Infectious Diseases	India	Pune	1	NT/D	Unknown		Bacteraemia
	Ponugoti	Journal of Medical Science And Clinical Research	India	Nellore	1	U/T	Unknown (6 months)		Sepsis with positive BC
	Sarangi	International Journal of Advances in Medicine	India	Bhubaneswar	1	U/T	01Nov12	30Apr14	Sepsis with positive BC
	Ting	Journal of Microbiology, Immunology and Infection	Republic of (Taiwan)	Taipei	1	U/T	01Jan02	31Dec11	CA bacteraemia, limited to 0-7 day-olds
	Tran	Journal of Perinatology	Vietnam	Da Nang	1	Maternity/ Paediatric	01Nov10	31Oct11	Sepsis with positive BC
2016	Abu	Medical Journal of Malaysia	Malaysia	Baru Selayang	1	U/T	01Jan01	31Dec11	CA bacteraemia excluding EOS
	Amin	International Journal of Pharmaceutical Sciences and Research	India	Vadodara	1	U/T	01Apr13	30Sep13	Sepsis with positive BC
	DeNIS	Lancet Global Health	India	Delhi	3	U/T	18Jul11	28Feb14	Sepsis with positive BC
	Jiang	Internal Medicine	China	Missing	1	Maternity/ Paediatric	01Jan08	31Dec12	Sepsis with positive BC
	Lu	Journal of Pediatrics and Child Health	China	Chongqing	1	Paediatric	01Jan90	31Dec14	Sepsis with positive BC
	Mahmood	Pakistan Journal of Medical and Health Sciences	Pakistan	Faisalabad	1	U/T	01Jan13	01Jan15	Bacteraemia
	Pandita	International Journal of Contemporary Pediatrics	India	Dehradun	1	U/T	01Jan13	30Jun15	Sepsis with positive BC
	Singh	European Journal of Pharmaceutical and Medical Research	India	Raipur	1	U/T	01Jan13	31Dec13	Sepsis with positive BC
	Thakur	Indian Journal of Medical Microbiology	India	Tanda	1	NT/D	01Apr12	31Mar13	Sepsis with positive BC

	Ullah	Archives of Iranian Medicine	Pakistan	Peshawar	1	U/T	01Jan12	31Dec15	Bacteraemia
2017	Dalal	International Journal of Research in Medical Sciences	India	Rohtak	1	U/T	01Jul10	30Sep13	Sepsis with positive BC
	Dong	BMC Pediatrics	China	Bengbu	1	NT/D	01Jan10	31Aug14	Sepsis with positive BC
	Ingale	International Journal of Contemporary Pediatrics	India	Pune	1	U/T	Unknown (1 year)		Sepsis with positive BC
	Kanodia	Journal of College of Medical Sciences – Nepal	Nepal	Dharan	1	U/T	01Jan14	31Dec14	Sepsis with positive BC
	Panigrahi	Journal of Perinatology	India	Multiple in area of Odisha	2	NT/D	01Apr02	31Mar05	Invasive bacterial infections
	Pavan	Journal of Family Medicine and Primary Care	India	Dindigul	1	NT/D	01Oct13	30Sep15	Sepsis with positive BC
	Roy	Journal of Postgraduate Medicine	India	New Delhi	1	U/T	01Jan11	31Dec14	Bacteraemia
	Sari	Asian Journal of Pharmaceutical and Clinical Research	Indonesia	Yogyakarta	1	U/T	01Jan14	31Dec15	Bacteraemia
2018	Dhaneria	Diseases	India	Ujjain	1	U/T	01Jun12	31Jan14	Nosocomial bacteraemia, including EONS and LONS
	Fox-Lewis	Emerging Infectious Diseases	Cambodia	Siem Reap	1	Paediatric	01Jan07	31Dec16	Invasive bacterial infections
	Jajoo	PloS One	India	Delhi	1	NT/D	01Jul11	31Jan15	Sepsis with positive BC
	Pokhrel	BMC Pediatrics	Nepal	Lalitpur	1	U/T	15Apr14	15Apr17	Sepsis with positive BC
	Wang	Journal of Tropical Pediatrics	China	Chongqing, Henan	2	U/T	01Jan03	31Dec13	Nosocomial bacteraemia
	Yadav	BMC Research Notes	Nepal	Kathmandu	1	Paediatric	01Apr15	30Sep15	Sepsis with positive BC
2019	Li	Medicine	China	Shanghai	1	U/T	01Jan13	31Aug17	Sepsis with positive BC

U/T hospital: University/Tertiary hospital; NT/D hospital: Non-teaching/District hospital
*Start year of data collection for all studies with exception of Lu *et al*, 2016 in the 2000s, end year for all studies in the 2000s.

© 2020 Bielicki JA et al. *JAMA Network Open.*

eTable 2. Information on Sample Processing Provided in Included Publications

Publication year, First author		Species identification	Antibiotic susceptibility testing method	Interpretive guidelines	Other comments
2014	Adhikari	Yes (Standard bacteriological techniques)	Yes (Disc diffusion)	Yes (CLSI M2-A9, 2006)	
	Anderson	No details provided (standard blood culture)	Yes (Disc diffusion)	Yes (CLSI M100-S20, 2010)	ESBL detection by cefpodoxime screening with confirmation by CLSI-recommended disc diffusion methods
	Javali	No details provided (standard blood culture)	Yes (Disc diffusion)	Yes (CLSI, 2008)	
	Khanal	Yes (Standard bacteriological techniques)	Yes (Disc diffusion)	Yes (CLSI M100-S16, 2007)	
	Methta	Yes (Standard bacteriological techniques)	Yes (Disc diffusion)	Yes (CLSI M100-S18, 2010)	Meropenem SIR based on imipenem susceptibility testing
	Mustafa	Yes (Standard bacteriological techniques)	Yes (Disc diffusion)	Yes (CLSI, not specified)	ESBL confirmation by phenotypic confirmatory test (ceftazidime/cefotaxime +/- clavulanate disc diffusion)
	Nayak	Yes (Standard bacteriological techniques)	Yes (Disc diffusion)	Yes (CLSI, not specified)	Use of control strains Meropenem SIR based on imipenem susceptibility testing
	Patel	Yes (BacT/ALERT, API)	Yes (automated API)	No details provided	
	Tudu	Yes (BacT/ALERT, API)	Yes (Disc diffusion)	Yes (CLSI, no specified)	Gentamicin SIR based on amikacin susceptibility testing, meropenem SIR based on imipenem susceptibility testing
	Venkatnarayan	No details provided	No details provided	No details provided	Gentamicin SIR based on amikacin susceptibility testing
2015	Agarwal	Yes (BacT/ALERT, Vitek II)	Yes (Disc diffusion)	Yes (CLSI M02-A11, 2012)	ESBL confirmed using CLSI-recommended disc diffusion methods,

© 2020 Bielicki JA et al. *JAMA Network Open.*

					MRSA detection using cefoxitin disc
	Ambade	Yes (Standard bacteriological techniques)	Yes (Disc diffusion)	Yes (CLSI, not specified)	
	Chapagain	No details provided	No details provided	No details provided	Gentamicin SIR based on amikacin susceptibility testing
	Dhanalakshmi	Yes (Standard bacteriological techniques)	Yes (Disc diffusion)	No details provided	
	Gupta	Yes (Standard bacteriological techniques)	Yes (Disc diffusion)	Yes (CLSI M100-S24, 2014)	Use of control strains
	Kamble	Yes (Standard bacteriological techniques)	Yes (Disc diffusion)	Yes (CLSI, not specified)	Extensive detail on testing for ESBL and Metallo-beta-lactamases provided<br>Meropenem SIR based on imipenem susceptibility testing
	Madavi	No details provided	No details provided	No details provided	Meropenem SIR based on imipenem susceptibility testing
	Marwah	Yes (Standard bacteriological techniques)	No details provided (standard methods)	Yes (CLSI, incorrect referencing)	Meropenem SIR based on imipenem susceptibility testing
	Muley	Yes (standard bacteriological techniques)	Yes (Disc diffusion)	Yes (CLSI M100-S21, 2011)	
	Ponugoti	Yes (standard bacteriological techniques)	Yes (Disc diffusion)	Yes (CLSI M2A7 Vol.20 No1 & 2, 2000)	Meropenem SIR based on imipenem susceptibility testing
	Sarangi	Yes (BacT/ALERT)	Yes (automated API)	No details provided	
	Ting	No details provided	No details provided	Yes (CLSI, not specified)	
	Tran	Yes (Standard bacteriological techniques)	Yes (Disc diffusion)	No details provided	Meropenem SIR based on imipenem susceptibility testing
2016	Abu	Yes (API/Vitek)	Yes (Disc diffusion)	Yes (CLSI M100-S24)	ESBL confirmation by phenotypic confirmatory test (ceftazidime/cefotaxime +/- clavulanate disc diffusion)
	Amin	Yes (Standard bacteriological techniques)	Yes (Disc diffusion)	Yes (CLSI, not specified)	Microbiology laboratory accredited by National Accreditation Board for

© 2020 Bielicki JA et al. *JAMA Network Open.*

					Testing and Calibration Laboratory in India
	DeNIS	Yes (Standard bacteriological techniques)	No details provided	Yes (CLSI M100-S21 & M100-S22 & M100-S23, 2011-2013)	Flowchart of sample handling provided in web-extra material
	Jiang	Yes (BacT/ALERT, API/Vitek)	Yes (Disc diffusion or Etests)	Yes (CLSI, not specified)	
	Lu	No details provided	No details provided	No details provided	Results recorded based on routine laboratory testing<br>Meropenem SIR based on imipenem susceptibility testing
	Mahmood	No details provided	No details provided	No details provided	Standard procedures for sample processing and interpretation
	Pandita	Yes (Bactec/API)	Yes (Disc diffusion)	Yes (CLSI M100-S21, 2011)	Meropenem SIR based on imipenem susceptibility testing
	Singh	Yes (Standard bacteriological techniques)	Yes (Disc diffusion)	Yes (CLSI M100-S18, 2008)	Gentamicin SIR based on amikacin susceptibility testing
	Thakur	Yes (Standard bacteriological techniques)	Yes (Disc diffusion)	Yes (CLSI M100-S21, 2011)	Use of control strains, MRSA screening using cefoxitin disc, ESBL screening using ceftazidime disc, confirmation of ESBL by double disc synergy test<br>Meropenem SIR based on imipenem susceptibility testing
	Ullah	Yes (Standard bacteriological techniques)	Yes (Disc diffusion)	Yes (CLSI, not specified)	Meropenem SIR based on imipenem susceptibility testing
2017	Dalal	No details provided (standard blood culture)	Yes (Disc diffusion)	No details provided	Meropenem SIR based on “carbapenem” susceptibility testing
	Dong	Yes (BacT/ALERT)	Yes (Disc diffusion)	No details provided	Additional information on species identification

© 2020 Bielicki JA et al. *JAMA Network Open.*

					and susceptibility testing provided in methods
	Ingale	Yes (Bactec/API)	Yes (Disc diffusion)	Yes (CLSI M100-S23, 2013)	Extensive detail on microbiological sample handling provided
	Kanodia	No details provided	Yes (Disc diffusion)	No details provided	
	Panigrahi	Yes (Bactec/API)	No details provided	Yes (CLSI M23-A2, 2001)	Extensive detail on microbiological sample handling provided<br>Meropenem SIR based on imipenem susceptibility testing
	Pavan	Yes (Bactec/API)	Yes (automated API)	No details provided	
	Roy	Yes (Standard bacteriological techniques)	Yes (Disc diffusion)	Yes (CLSI M100-S19, 2009)	Extensive detail on microbiological sample handling provided; ESBL confirmation by phenotypic confirmatory test (ceftazidime/cefotaxime +/- clavulanate disc diffusion); Use of control strains; MRSA screening using oxacillin disc<br>Gentamicin SIR based on amikacin susceptibility testing
	Sari	Yes (Vitek)	Yes (Disc diffusion)	No details provided	
2018	Dhaneria	Yes (Standard bacteriological techniques)	Yes (Disc diffusion, confirmation using Vitek 2)	Yes (CLSI M100-S21, 2011)	Extensive detail on microbiological sample handling provided<br>Meropenem SIR based on imipenem susceptibility testing
	Fox-Lewis	Yes (Standard bacteriological techniques)	Yes (Disc diffusion or Etests)	Yes (CLSI, 2012)	Meropenem SIR based on imipenem susceptibility testing
	Jajoo	Yes (Bactec/Vitek)	No details provided	Yes (CLSI M100-S21 & M100-S22 & M100-S23, 2011-2013)	Aminoglycosides and carbapenems grouped in susceptibility reporting
	Pokhrel	Yes (Bactec)	Yes (Disc diffusion)	Yes (CLSI M100-S24, 2014)	

© 2020 Bielicki JA et al. *JAMA Network Open.*

	Wang	Yes (Vitek/API)	Yes (Disc diffusion)	Yes (CLSI, 2015)	Use of control strains, ESBL screening using ceftazidime disc, confirmation of ESBL by combination discs Meropenem SIR based on imipenem susceptibility testing
	Yadav	Yes (Standard bacteriological techniques)	Yes (Disc diffusion)	Yes (CLSI M100-S23, 2014)	Use of control strains
2019	Li	No details provided	Yes (Disc diffusion)	Yes (CLSI, not specified)	

CLSI: Clinical and Laboratory Standards Institute; ESBL: extended-spectrum beta-lactamases; MRSA: methicillin-resistant *Staphylococcus aureus*

© 2020 Bielicki JA et al. *JAMA Network Open.*

eTable 3. Relative Incidence of Bacteria in Included Studies

Publication year, First author		Total bacterial isolates	Bacteria reported in studies (% incidence within study shown)																						% accounted for by 7 target species	% accounted for by 7 target species excluding CONS
			Acinetobacter spp.[1]	*Burkholderia* spp.	*Citrobacter* spp.	CONS	*E. coli*	*Enterobacter* spp.[2]	*Enterococcus* spp.	*H. influenzae*	*Klebsiella* spp.[3]	*L. monocytogenes*	*Morganella* spp.	*N. meningitidis*	*Proteus* spp.	*Pseudomonas* spp.[4]	*S. agalactiae*	*S. aureus*	*S. pneumoniae*	*S. pyogenes*	*Salmonella* spp.	*Serratia* spp.[5]	Other streptococci	Others		
2014	Adhikari	94				57	27				4					1		11							43	100
	Anderson*	75	3	3			11	5	4		12	1			1	1	3	49	3	3	1				85	85
	Javali	32	9			34	13				19							9					9	6	50	77
	Khanal	61				77	10		4		2							7							23	100
	Mehta	169	5		2	9	4		5		14					5		56							89	98
	Mustafa	62				11	23				35					7		24							89	100
	Nayak	67	20		3	5	4		3		31					4		20							82	96
	Patel*	249	5			12	10	10	2		47					6		1						6	81	93
	Tudu	22				5	9		18		9	5						55							91	95
	Venkatnarayan	15				13	20									13	7	47							80	92
2015	Agarwal*	34	15			9	24	3			27							21							90	100
	Ambade	119	6			10	14				35					13		22							90	100
	Chapagain	30	7			7		3					3					80							90	97
	Dhanalakshmi	41				10	10				68				5	7									85	94
	Gupta	325	12		5	13	8	2	8		13					20		20							83	94
	Kamble	71	14		1	17	7	1	6		23					21		7	1						79	98
	Madavi	103	19		1	16	6	1	7		22					17		5	<1		1			5	77	92
	Marwah	167	15				7				15							47						16	84	84
	Muley	48	10			6	17				35					8		23							93	100
	Ponugoti	188	2		3	15	22	19	1		25					2		12							83	97
	Sarangi	74	3	5		62	11	8										8			3				30	79
	Ting*	36					31				3	8					42							17	34	34
	Tran	75	23			31	3	8			24					5		5					1		68	99
2016	Abu*	29					21			3	3					3	21	35		3			7	3	62	63
	Amin	101	23			4	12		13		28					8		13							97	100
	DeNIS	998	22			15	14	4	6		17					7	1	12						1	82	98
	Jiang*	131	1	1		43	19	6	5		13	3					1	6					1	1	50	88
	Lu*	929	3			26	14	3	7		12	2				4		6					5	18	49	66

© 2020 Bielicki JA et al. *JAMA Network Open.*

	Mahmood	341					48		<1		17				9			26	<1						91	91
	Pandita	124	6		6	26	11	6	2		27					3		8					1	4	63	85
	Singh	141				5	27		4		50					8		7							96	100
	Thakur*	188	1		4	19	5	5			10					15	2	40							76	93
	Ullah	1534				2	53				7				6	13		20			<1				93	94
2017	Dalal	356	15			4	12	1	2		4					47		12							93	100
	Dong*	93				73	6	2	1		11	1				1		2					1	1	23	88
	Ingale	48	13			25	2	6	10		29					13		2							75	100
	Kanodia	327	14		1	2	3	3	4		1					6		62					3		93	96
	Panigrahi*	56					14		2		52							20						12	88	88
	Pavan	28					11				21					4		36					4	24	72	72
	Roy*	2112	21			21	8		5		8							25						12	67	85
	Sari	225	9	9		28		9			22					14						9			54	75
2018	Dhaneria*	46				17	11				24				9	13		21						5	69	83
	Fox-Lewis*	185	9	2			14	10		1	32			1		3		18	1	9	1				86	85
	Jajoo	300	15	4	1	14	11	8	5	<1	18		<1		1	1	1	6	1	1	<1	<1	<1	12	64	75
	Pokhrel*	69	12			20	4	19			33					3		2				4	2	2	73	90
	Wang	571				39	18	3			17							5	2					16	43	70
	Yadav	59	12		2	10	7	10			15					7		36			2				87	96
2019	Li*	339	<1			44	10	1	6		9	<1				5	6	5				1	3	10	36	64

**a priori* exclusion of contaminants with or without definitions for exclusion process provided

[1]includes *A. baumannii, A. lwoffi*

[2]includes *E. cloacae*

[3]includes *K. pneumoniae, K. ornithinolytica, K. oxytoca, K. ozaenae*

[4]includes *P. aeruginosa*

[5]includes *S. marcescens, S. rubidaea*

© 2020 Bielicki JA et al. *JAMA Network Open.*
